# Optimization of Naringin Extraction, Synthesis of Dihydrochalcone and Its Effects on Reducing Blood Lipid Levels In Vitro

**DOI:** 10.3390/molecules29235778

**Published:** 2024-12-06

**Authors:** Xiaolei Yu, Haowei Wu, Lei Zhang, Dongliang Fei

**Affiliations:** 1 Meat Processing and Safety Control Professional Technology Innovation Center, Jinzhou Medical University, Jinzhou 121000, China; yuxiaolei@jzmu.edu.cn (X.Y.); 15153437780@163.com (H.W.); 2 MOE Key Laboratory for Nonequilibrium Synthesis and Modulation of Condensed Matter, School of Physics, Xi’an Jiaotong University, Xi’an 710049, China

**Keywords:** naringin, extraction optimization, catalysis, naringin dihydrochalcone, in vitro hypolipidemic, response surface methodology

## Abstract

Response surface methodology (RSM) was used to optimize the extraction process of naringin. The central component design included three parameters of extraction, namely temperature (X_1_), solid–liquid ratio (X_2_), and extraction time (X_3_). The optimum extraction temperature was 67 °C; the ratio of feed to solvent was 54:1 mL/g, and the extraction time was 2.8 h. According to the best extraction conditions, naringin was processed to verify the accuracy of the model. Five parallel experiments were set up, and a yield of 3.248% naringin was obtained, which was equivalent to the predicted yield of 3.256%. Naringin was purified to obtain naringin-refined products using DM101 macroporous adsorption resin. Naringin dihydrochalcone was synthesized following catalytic hydrogenation of purified naringin. The structures of naringin and naringin dihydrochalcone were determined via Fourier infrared spectrometer and nuclear magnetic resonance spectrometry. In vitro determination of the lipid-lowering activity of naringin dihydrochalcone was also conducted. Further focusing on HepG2 cells, a high cholesterol-induced high-fat HepG2 cell model was established. We measured the effects of different concentrations of naringin dihydrochalcone on intracellular lipids in denatured HepG2 cells and further validated the lipid-lowering effect of naringin at the cellular level. The results showed that naringin dihydrochalcone has a potential application in functional foods for lowering blood lipids.

## 1. Introduction

In recent years, hyperlipidaemia has become a global public health problem and has seriously affected people’s physical and mental health and the quality of their life. Hyperlipidaemia, a condition characterized by high lipid levels in the blood owing to abnormal lipid metabolism, is often associated with induced cardiovascular diseases, such as hypertension, coronary heart disease, and atherosclerosis. With great changes in people’s lifestyles, the incidence of hyperlipidaemia has dramatically increased in recent years. Related medical research has confirmed that 75% of every 100 patients with hyperlipidaemia have varying degrees of risk of cardiovascular and cerebrovascular diseases. At present, statins are widely used in the treatment of hyperlipidaemia. The long-term use of these hypolipidemic drugs leads to gastrointestinal discomfort, rash, myalgia, and greater side effects of liver transaminase. Therefore, the intervention of food nutritional components in diet-induced hyperlipidaemia has become a new research hotspot in the field of food nutrition [1].

The scientific name of pomelo is *Citrus maximum* (Burm.) Merr. Pomelo is the mature fruit of the citrus plant in the Rutaceae family and is one of the main fruit resources in China. The outer skin of pomelo is relatively thick, accounting for about 30% to 60% of the total amount of pomelo. Whether consumed fresh or in products such as fruit juice and jam, a large quantity of by-products, such as skin and residue, are produced, and the utilization rate of these by-products is extremely low. Up to 90% of these by-products are discarded as garbage, which seriously wastes resources and also causes substantial environmental pollution [2,3]. It has been found that naringin dihydrochalcone has a certain effect on diabetes [4]. In addition, the effective group of naringin dihydrochalcone with antioxidant effect—2,6-dihydroxyacetophenone structure—is realized by eliminating peroxide and hydroxyl radical [5]. At present, studies on the hypolipidemic effect of naringin dihydrochalcone are limited. A previous study by our research group showed that naringin exerts a blood lipid lowering effect [6]. This study used an ethanol heating extraction method with extraction temperature, feed-to-solvent ratio, extraction time, and other main parameters. Response surface methodology combined with a single-factor experimental strategy was used to ultimately determine the optimal process conditions for the method of naringin extraction. Compared with traditional extraction methods, the extraction solvent ethanol is non-toxic and suitable for large-scale industrial production. In this study, naringin monomer was extracted from pomelo peel, and catalysed hydrogenation was used to prepare naringin dihydrochalcone. The ability of naringin dihydrochalcone to bind sodium glycine cholate and sodium bovine cholate was evaluated by simulating the gastrointestinal environment in vitro so as to evaluate its lipid-lowering activity. HepG2 cells, a human liver cancer cell line, are commonly used by medical researchers worldwide to study liver lipid metabolism [7,8]. Therefore, this study further focused on HepG2 cells and established a high-fat HepG2 cell model induced by high cholesterol [9,10,11]. The effects of different concentrations of naringin dihydrochalcone on the intracellular lipids of denatured HepG2 cells were measured, and the lipid-lowering effect of naringin dihydrochalcone was further validated at the cellular level in order to provide theoretical basis for the development of naringin dihydrochalcone functional food. Through in vitro lipid-lowering experiments, this article explores the lipid-lowering effects of naringin dihydrochalcone, laying the foundation for the development of naringin dihydrochalcone series functional foods.

## 2. Results and Discussion

### 2.1. Determination of Optimum Extraction Conditions of Naringin

#### 2.1.1. Naringin Standard Curve

The standard curve of naringin is shown (Supplementary Materials Figure S1).

Regression equations were obtained with naringin concentrations of 0, 0.005, 0.010, 0.015, 0.020, 0.025, and 0.030 mg/mL as the *x*-axis (1):

(1) y=36.391x−0.0183

R^2^ = 0.9991; linear range from 0 to 0.0288 mg/mL; good linear relationship. Naringin content can be calculated based on this standard curve.

#### 2.1.2. Single-Factor Experimental Results

The effect of extraction temperature on naringin extraction yield is shown (Supplementary Materials Figure S2). From Figure S2A, it is evident that with an increase in temperature, molecular movement intensified, resulting in the dissolution of naringin and a continuous increase in its extraction yield, which reached a maximum at 65 °C. When the temperature was increased further, the naringin structure was destroyed and degraded; thus, the extraction yield decreased significantly [12]. Further, Figure S2B shows that as the feed-to-solvent ratio gradually increases, the extraction yield of naringin gradually increases; when the feed to solvent ratio is below 45:1 mL/g, the low activation energy and incomplete dissolution of naringin may lead to a lower extraction yield of naringin. As the feed-to-solvent ratio gradually increases, the permeability of pomelo peel tissue increases, and the activation energy of naringin increases, which is conducive to the diffusion of naringin into the solvent. The extraction yield increases rapidly. When the feed-to-solvent ratio reaches 50:1 mL/g, the extraction yield of naringin basically decreases [13]. This may be because, as the ratio of feed to liquid increases, a larger concentration difference is formed inside and outside the pomelo peel cells, which enhances the diffusion ability inside the pomelo peel cells and leads to a large amount of naringin dissolution. However, when the feed-to-solvent ratio is excessive, the diffusion of naringin in the extraction solution tends to reach an equilibrium state. Therefore, the optimal feed-to-solvent ratio was 50:1 mL/g [14]. As shown in Figure S2C, with an increase in extraction time, the extraction yield of naringin increased gradually, peaking at 2.5 h, after which it dropped sharply. This may be due to prolonged extraction time, evaporation of the extraction solution, increased material viscosity, slow diffusion on the solid surface, or reduced driving force of the solvent due to equilibrium or solubility limitation of naringin at specific temperatures, resulting in a slow increase in extraction yield. Therefore, 2.5 h was identified as the optimal extraction time.

#### 2.1.3. Response Surface Optimization Test Results and Analysis of Variance

The process and results of the response surface optimisation tests are listed in Table 1. Using Design-Expert 8.0.6 software, model fitting analysis was performed on the test results shown in Table 1, considering the extraction temperature (X_1_), feed-to-solvent ratio (X_2_), and extraction time (X_3_) as independent variables and the extraction yield (Y) as the response value. Thus, the following regression Equation (2) was obtained:

Y = 31.80 + 0.44 X_1_ + 0.75 X_2_ + 1.13 X_3_ − 0.18 X_1_ X_2_ − 0.088 X_1_ X_3_ + 0.38 X_2_ X_3_ − 0.30 X_1_^2^ − 0.58 X_2_^2^ − 1.04 X_3_^2^ (2)

Via the analysis of variance (ANOVA), we further determined the quality of the model and clarified the relationship between various parameters. The misfit error obtained (*p* = 0.105 > 0.05), as shown in Table 2, indicated a good data fitting effect, with the primary and secondary terms reaching a highly significant level and the interaction terms AB and BC showing significance. Further, the model design was reasonable, and the response surface model could accurately reflect the relationship between naringin yield and the extraction conditions. The accuracy test for the regression equation also showed that the coefficient of determination, R^2^, of the equation model was 0.9948, which is indicative of the highly significant performance of the model. Furthermore, R^2^adj = 0.9881 could explain the 98.81% response value variation in the experiment, indicating that the experimental model had good fit with real data and has practical guidance significance. The model can be used to interpret the effect of independent variables on the extraction of the selected metabolite. Therefore, the model can be used to analyse and predict the optimal naringin extraction process [15].

#### 2.1.4. Response Surface Graphic Analysis

**Figure 1:**
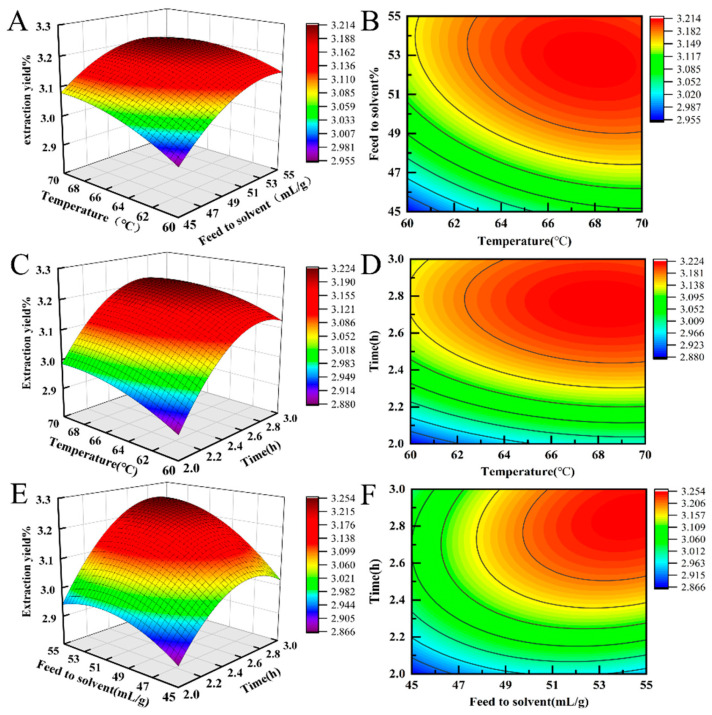
3D response surface plots (**A**,**C**,**E**) and contour plots (**B**,**D**,**F**) showing the interaction effects on the naringin extraction yield; (**A**,**B**) exaction-temperature- and exaction-feed-to-solvent ratio; (**C**,**D**), exaction temperature and exaction time; (**E**,**F**),exaction feed to solvent ratio and exaction time.

The response surface analysis of the extraction temperature, feed-to-solvent ratio, and extraction time is shown in Figure 1. Observing the steepness of the response surfaces using a 3D graph showed a steeper slope and a higher degree of inclination, indicating that the experimental results shown on the vertical axis were more sensitive to the factors shown on the corresponding horizontal axis and that the interaction between the two factors had a stronger impact on the experimental results. From the perspective of the contour map transformed from the 3D graph, a saddle-shaped contour indicated a significant interaction. The smaller the eccentricity of the ellipse, and the closer it was to a circle, the less significant the interaction. Further, the steeper the response surface curve, the more important was the impact. These criteria have been widely applied in other studies. Figure 1 also validated the ANOVA results. Among the three factors, extraction time showed the most significant impact, followed by the feed-to-solvent ratio and extraction temperature. Further, we also found that among the pairwise interaction surfaces of the various factors, the longitudinal span of the interaction between the feed-to-solvent ratio and extraction time was the largest. Furthermore, the eccentricity of the contour ellipse was the largest. This indicated that the interaction between the two factors had the most significant impact on the naringin extraction yield [16]. Additionally, the longitudinal span of the interaction between extraction temperature and the feed-to-solvent ratio was the larger, and similarly, the eccentricity of the contour ellipse was the largert. Our results also indicated that the longitudinal span of the interaction between extraction temperature and extraction time was the smaller, and the eccentricity of the corresponding contour ellipse was also the smaller. We also noted that the trend of effect of the interactions between extraction temperature, liquid–solid ratio, and extraction time on naringin extraction yield was BC > AB > AC, consistent with the results of ANOVA. This also indicated that the response surface analysis method could be used to analyse and predict the optimal naringin extraction process conditions.

#### 2.1.5. Determination of Optimal Extraction Conditions

Based on the response surface of each factor, it could be concluded from the highest point of the response surface that some extreme values exist for each factor within the set experimental range. According to the regression model prediction, the optimal naringin extraction conditions were determined to be as follows: temperature, 66.93 °C; feed-to-solvent ratio, 54:1 mL/g; and extraction time, 2.8 h. Under these conditions, the predicted yield of naringin was 3.256%. Further, considering the actual situation, the optimal process conditions were adjusted to a temperature, feed-to-solvent ratio, and extraction time of 67 °C, 54:1 mL /g, and 2.8 h, respectively. The raw materials were processed under these optimal extraction conditions in order to verify the accuracy of the model. To this end, a total of five parallel experiments were set up, and the average yield of naringin was 3.248%. Thus, the extraction and determination model showed high accuracy, consistent with the predicted value. This indicated that the extraction method was feasible [17].

### 2.2. Structural Analysis of Naringin

#### 2.2.1. IR Spectrum Analysis

The IR analysis results for our refined naringin products are shown (Supplementary Materials Figure S3).

Figure S3 shows that the strong and wide absorption peaks at 3180–3680 cm^−1^ were attributed to the alcohol hydroxyl groups and multiple phenolic hydroxyl groups in naringin. The absorption peaks at 3000–3300 cm^−1^ were attributed to the C-H stretching vibration on the benzene ring. Those at 2850–2950 cm^−1^ were attributed to the C-H bond stretching vibration on the saturated carbon in the structure. Generally, the carbonyl stretching vibration absorption peak of flavonoids is located at approximately 1650 cm^−1^, while that of dihydroflavonoids is located at approximately 1695 cm^−1^. However, the hydroxyl group at position 5 in naringin forms intramolecular hydrogen bonds with the carbonyl group, and this results in the absorption peak shifting towards low frequencies. Finally, the carbonyl stretching vibration at position 4 in naringin appeared at 1647 cm^−1^, and the peaks at 1581, 1518, and 1447 cm^−1^ were attributed to the C=C stretching vibrations of the aromatic ring in the structure. Those at 1369 and 1296 cm^−1^ were attributed to the in-plane bending vibration of the methylene group in the structure and the multiple absorption peaks at 1208–1035 cm^−1^ were attributed to the C-O stretching vibrations of the aromatic ether and fatty ether bonds in the structure. Additionally, the glycosidic bond absorption peak at 885 cm^−1^ indicated the presence of the β-D-pyranoside glycosidic bond. The absorption peak at 818 cm^−1^ could be attributed to the C-H out-of-plane bending vibration of the B-ring aromatic ring para-substituted structure. By analyzing the spectral data obtained, it was determined that the extract was naringin.

#### 2.2.2. NMR Spectrum Analysis

**Figure 2:**
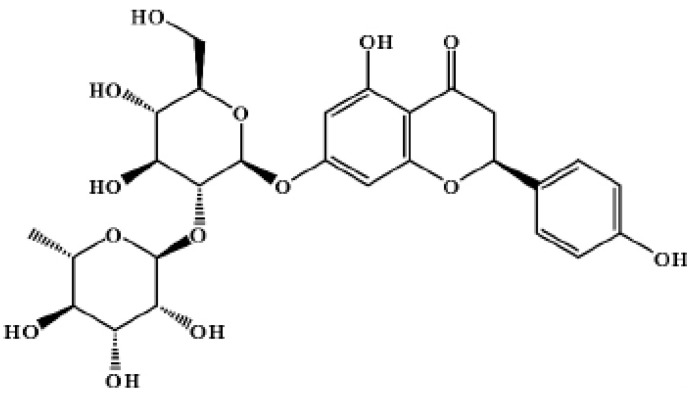
Structural formula of naringin.

The structural formula of naringin is shown as Figure 2.

The NMR analysis of our refined naringin products is shown (Supplementary Materials Figures S4–S7).

As shown in Figures S4–S7, the ^1^H NMR and ^13^C NMR spectra of our refined naringin products are highly consistent with those of the standard.

Naringin sample

^1^H NMR (500 MHz, Methanol-d4) δ 7.31 (d, *J* = 8.2 Hz, 2H), 6.83 (d, *J* = 8.2 Hz, 2H), 6.17 (dd, *J* = 6.7, 2.4 Hz, 2H), 5.33 (dt, *J* = 13.0, 3.2 Hz, 1H), 5.27 (dd, *J* = 4.3, 1.7 Hz, 1H), 5.09 (dd, *J* = 9.3, 7.4 Hz, 1H), 4.02–3.95 (m, 1H), 3.90 (ddd, *J* = 17.2, 11.2, 3.6 Hz, 2H), 3.66 (dddd, *J* = 25.8, 17.5, 8.6, 6.0 Hz, 4H), 3.50–3.37 (m, 3H), 3.14 (ddd, *J* = 17.3, 13.0, 8.3 Hz, 1H), 2.74 (dt, *J* = 17.2, 2.6 Hz, 1H), 1.31 (d, *J* = 6.2 Hz, 3H).

^13^C NMR (126 MHz, Methanol-d4) δ 197.22, 165.07, 163.26, 129.30, 127.89, 127.77, 115.03, 103.53, 101.18, 101.12, 98.00, 97.94, 96.53, 95.41, 79.30, 79.24, 77.77, 77.64, 77.50, 76.66, 72.54, 70.77, 69.80, 68.62, 60.87, 48.03, 47.86, 47.69, 47.52, 47.35, 42.77, 42.43, 16.90.

Naringin standard

^1^H NMR (500 MHz, Methanol-d4) δ 7.31 (d, *J* = 8.2 Hz, 2H), 6.83 (d, *J* = 8.2 Hz, 2H), 6.17 (dd, *J* = 6.7, 2.4 Hz, 2H), 5.33 (dt, *J* = 13.0, 3.2 Hz, 1H), 5.27 (dd, *J* = 4.3, 1.7 Hz, 1H), 5.09 (dd, *J* = 9.3, 7.4 Hz, 1H), 4.02–3.95 (m, 1H), 3.90 (ddd, *J* = 17.2, 11.2, 3.6 Hz, 2H), 3.66 (dddd, *J* = 25.8, 17.5, 8.6, 6.0 Hz, 4H), 3.50–3.37 (m, 3H), 3.14 (ddd, *J* = 17.3, 13.0, 8.3 Hz, 1H), 2.74 (dt, *J* = 17.2, 2.6 Hz, 1H), 1.31 (d, *J* = 6.2 Hz, 3H).

^13^C NMR (126 MHz, Methanol-d4) δ 197.22, 165.07, 163.26, 129.30, 127.89, 127.77, 115.03, 103.53, 101.18, 101.12, 98.00, 97.94, 96.53, 95.41, 79.30, 79.24, 77.77, 77.64, 77.50, 76.66, 72.54, 70.77, 69.80, 68.62, 60.87, 48.03, 47.86, 47.69, 47.52, 47.35, 42.77, 42.43, 16.90.

The IR and NMR (Supplementary Materials Figures S3–S6) of our refined naringin products were shown and compared with the standard naringin or standard library [18]. Structural formula of naringin was shown in Figure 2.

### 2.3. Structural Analysis of Naringin Dihydrochalcone

#### 2.3.1. IR Spectrum Analysis

The IR analysis results for our refined naringin products are shown (Supplementary Materials Figure S8) [19].

The strong and wide absorption peaks at 3000–3680 cm^−1^ were attributed to the alcohol and phenolic hydroxyl groups in naringin dihydrochalcone. The absorption peaks at 2840–2980 cm^−1^ were attributed to the C-H bond stretching vibration on the saturated carbon in the structure. Further, the peak at 1630 cm^−1^ was attributed to the carbonyl stretching vibration absorption peak in naringin dihydrochalcone, while the peaks at 1520, 1440, and 1390 cm^−1^ were attributed to the C=C stretching vibrations of the aromatic ring in the structure. The multiple absorption peaks at 1200–1060 cm^−1^ were attributed to the C-O stretching vibrations of aromatic ether and fatty ether bonds in the structure, and the absorption peak at 820 cm^−1^ was attributed to the C-H out-of-plane bending vibration of the B-ring aromatic ring para-substituted structure.

#### 2.3.2. NMR Spectrum Analysis

**Figure 3:**
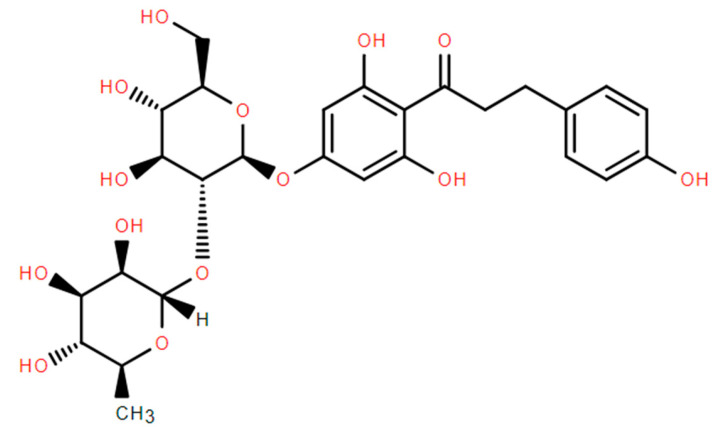
Structural formula of naringin dihydrochalcone.

The structural formula of naringin dihydrochalcone is shown as Figure 3.

The NMR analysis of our refined naringin dihydrochalcone products is shown (Supplementary Materials Figures S9–S12).

As shown (Supplementary Materials Figures S9–S12), the ^1^H NMR and ^13^C NMR spectra of our refined naringin dihydrochalcone products are highly consistent with those of the standard [20].

Naringin dihydrochalcone sample

^1^H NMR (500 MHz, Methanol-d4) δ 7.06 (d, *J* = 8.2 Hz, 2H), 6.72 (d, *J* = 8.0 Hz, 2H), 6.07 (s, 2H), 5.29 (d, *J* = 1.7 Hz, 1H), 5.04 (d, *J* = 7.5 Hz, 1H), 4.02–3.87 (m, 3H), 3.73 (dd, *J* = 12.1, 5.3 Hz, 1H), 3.69–3.58 (m, 3H), 3.45 (dtd, *J* = 16.7, 9.6, 8.6, 5.3 Hz, 3H), 3.33–3.26 (m, 2H), 2.87 (t, *J* = 7.8 Hz, 2H), 1.34 (d, *J* = 6.3 Hz, 3H).

^13^C NMR (126 MHz, Methanol-d4) δ 205.68, 164.00, 163.20, 132.49, 128.97, 114.78, 101.08, 97.93, 94.90, 77.64, 77.51, 76.72, 72.61, 70.83, 70.76, 69.86, 68.56, 60.95, 46.10, 29.82, 16.89.

Naringin dihydrochalcone standard

^1^H NMR (500 MHz, Methanol-d4) δ 7.06 (d, *J* = 8.4 Hz, 2H), 6.72 (d, *J* = 8.2 Hz, 2H), 6.07 (d, *J* = 1.8 Hz, 2H), 5.29 (s, 1H), 5.04 (d, *J* = 7.4 Hz, 1H), 4.04–3.89 (m, 3H), 3.73 (dd, *J* = 12.1, 5.3 Hz, 1H), 3.71–3.58 (m, 3H), 3.51–3.38 (m, 3H), 3.30 (ddd, *J* = 8.8, 6.8, 2.3 Hz, 2H), 2.86 (t, *J* = 7.8 Hz, 2H), 1.34 (dd, *J* = 6.2, 1.6 Hz, 3H).

^13^C NMR (126 MHz, Methanol-d4) δ 205.70, 163.98, 163.18, 132.49, 128.98, 114.80, 101.07, 97.93, 94.91, 77.63, 77.51, 76.71, 72.62, 70.83, 70.76, 69.87, 68.56, 60.95, 46.10, 29.81, 16.91.

The IR and NMR (Supplementary Materials Figures S7–S11) of our refined naringin dihydrochalcone products were shown and compared with the standard naringin dihydrochalcone standard library [21].

### 2.4. Cholate Standard Curve

The cholate standard curve is shown (Supplementary Materials Figures S13 and S14).

It can be seen from Figure S13 that the regression equation of the standard curve of sodium glycocholic acid is y = 0.91627 + 1.528x, R^2^ = 0.99834 with a strong linear relationship.

It can be seen from Figure S14 that the regression equation of the standard curve of sodium taurocholate is y = 1.01167 + 1.12x, R^2^ = 0.9991, with a strong linear relationship.

### 2.5. The Binding Ability of Naringin Dihydrochalcone to Cholate

**Figure 4:**
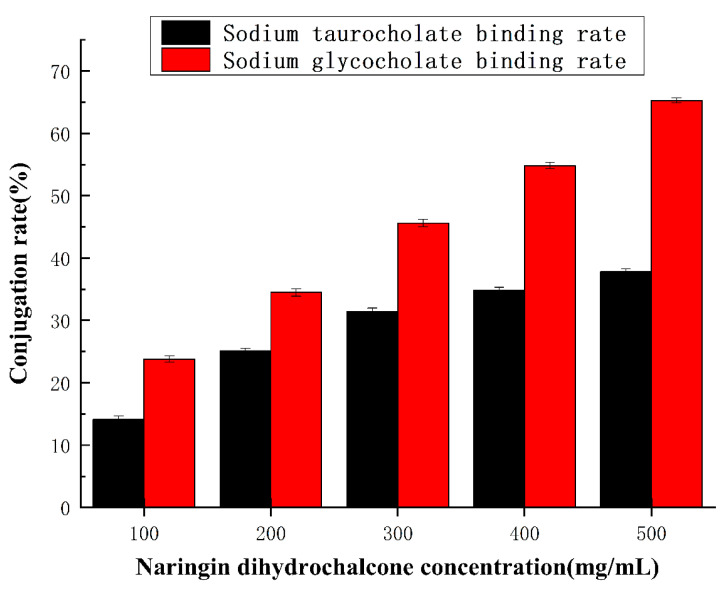
Binding capacity of naringin dihydrochalcone to cholate.

Bile acid salts are mainly concentrated in the bile of humans and animals and play an important role in the metabolism and absorption of lipids, cholesterol, fat-soluble vitamins, and other substances. Based on a simulated gastrointestinal environment, the in vitro lipid-lowering effect of naringin dihydrochalcone was evaluated by detecting its binding ability with two typical bile acid salts. As seen in Figure 4, with an increase in naringin dihydrochalcone concentration, the binding rate of sodium glycocholic acid and sodium taurocholate increases significantly (*p* < 0.05). When the concentration of naringin was 0.5 mg/mL, the binding rate of naringin dihydrochalcone and sodium glycocholic acid was 65.29 ± 0.36%, and that of naringin dihydrochalcone and sodium taurocholate was 37.84 ± 0.24%, indicating that the binding capacity of naringin dihydrochalcone and sodium glycocholic acid was stronger than that of sodium taurocholate. This may be related to the spatial structure of naringin dihydrochalcone molecules, and the specific reasons need to be further confirmed. Therefore, the higher the binding rate of naringin dihydrochalcone with sodium glycocholic acid and sodium taurocholate, the higher the binding capacity of sodium cholate, the stronger the blood lipid-lowering function, and the more significant the blood lipid-lowering effect [22,23]. The in vitro lipid-lowering test method is simple, has a short cycle, and has relatively few influencing factors. One of the important methods for identifying in vitro lipid-lowering activity is to determine the binding ability of the sample to bile salts. Bile acids are divided into free and bound bile acids, which are synthesized by the liver and flow into the intestine with bile secretions in the bile ducts. Free bile acids undergo passive reabsorption, combined with active reabsorption, to form a complete hepatic intestinal circulation system and regulate the bile acid content in the body to maintain a balanced and stable level. At present, there are few studies on the in vitro lipid-lowering effect of naringin dihydrochalcone. Therefore, by simulating the gastrointestinal environment and based on the binding effect of naringin dihydrochalcone on glycylcholic acid and bovine bile acid, the lipid-lowering effect of naringin dihydrochalcone is explored in order to provide an experimental basis for further research on the active function of naringin dihydrochalcone [24,25].

### 2.6. MTT Experimental Results

**Figure 5:**
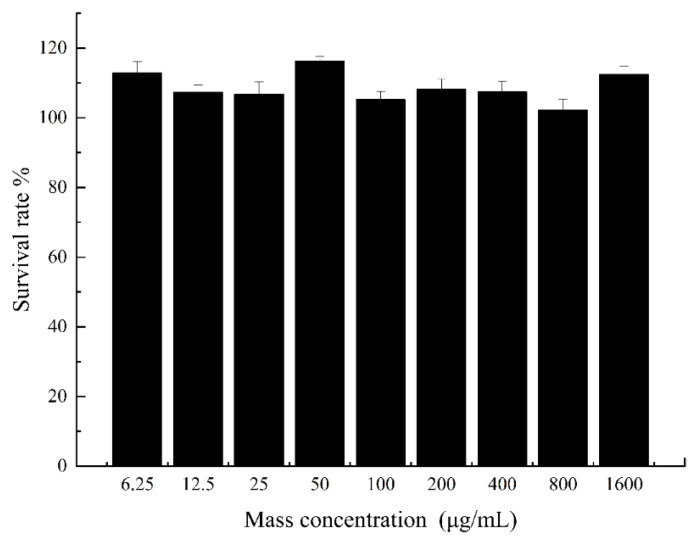
Effect of naringin dihydrochalcone on the survival of Hep G2 liver cancer cells.

The inhibitory effects of nine concentrations of naringin dihydrochalcone on HepG2 liver cancer cells are shown in Figure 5, which shows that at mass concentrations in the 6.25–1600 μg/mL range, the survival rate of the HepG2 cells is greater than 99.00%. This indicates that within this concentration range, naringin dihydrochalcone has no effect on the survival rate of HepG2 cells, and there is no significant difference compared to the control group (*p* > 0.05). Thus, subsequent experiments could be conducted using samples with concentrations in this range.

### 2.7. Establishment of the High-Fat Cell Model

**Figure 6:**
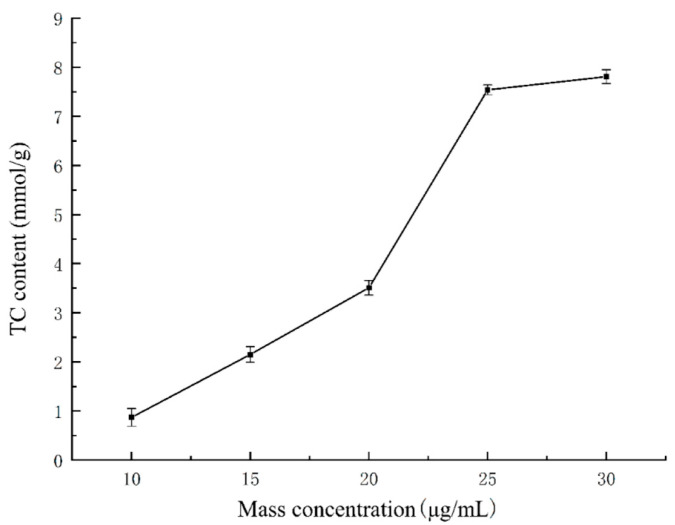
Effect of different concentrations of cholesterol on the total cholesterol content of HepG2 cells.

The construction of the high-cholesterol cell model using HepG2 liver cancer cells is shown in Figure 6. From this figure, it is evident that when the mass concentration of cholesterol was less than 25 μg/mL, the content of cholesterol in the cells increased with increasing cholesterol concentration. However, cholesterol mass concentrations above 25 μg/mL did not result in any significant increase in cholesterol content (*p* > 0.05). Further, when the mass concentration of cholesterol was 25 μg/mL, the corresponding cholesterol content was 7.41 ± 0.12 mmol/L, higher than the normal level. This observation indicated that with a cholesterol mass concentration of 25 μg/mL, the high-cholesterol cell model was successfully constructed.

### 2.8. Effects of Naringin Dihydrochalcone on TC, TG, LDL-C, and HDL-C Levels in HepG2 Cells

**Figure 7:**
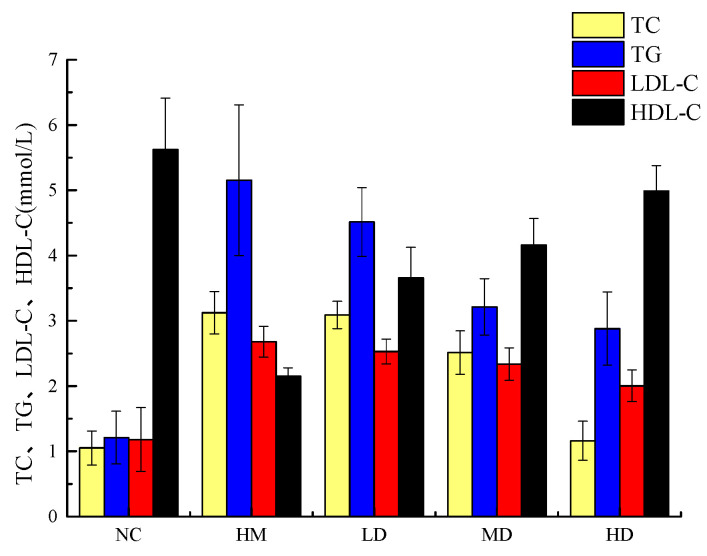
Effect of naringin dihydrochalcone on blood lipid levels in HepG2 cells (normal control, NC; hyperlipidaemia model group, HM; high-dose group, HD; medium-dose group, MD; low-dose group, LD).

Excessive fat intake can produce a large amount of free fatty acids, leading to the accumulation of triglycerides and the secretion of related lipoproteins in the body, ultimately leading to an increase in blood lipid levels [26]. The triglycerides in the human body are mainly biosynthesized into non-lipid substances by liver cells and adipocytes. HepG2 cells retain the normal biochemical functions of natural liver cells and are therefore widely used in the study of liver triglyceride synthesis ability [27,28]. In order to further investigate whether naringin dihydrochalcone has the potential to reduce blood lipids, we treated HepG2 cells with cholesterol, simulated a high-fat environment, established a high-cholesterol cell model, and then subjected them to naringin dihydrochalcone treatment. The effects of naringin dihydrochalcone on blood lipid levels treatment in HepG2 cells were investigated [29,30,31,32]. Cells in the normal control and high-cholesterol model groups were treated with different concentrations (10, 20, and 40 mg/kg) of naringin dihydrochalcone to study the effects of this agent on TG and TC levels in Hep G2 cells. The changes in TG content in the cells are shown in Figure 7. Within the 10–40 μg/mL concentration range, the TC and TG contents of the cells decreased with increasing naringin dihydrochalcone concentration. Further, compared with the blank group, the model group showed significantly higher TC, TG, and LDL-C levels (*p* < 0.01), while the levels of HDL-C were significantly reduced (*p* < 0.01). Further, compared with the model group, the low-, medium-, and high-dose naringin dihydrochalcone groups showed 20.36, 33.77, and 68.22% decreases in TC levels; 21.42, 41.78, and 55.44% decreases in TG levels; and 11.65, 20.15, and 28.71% decreases in LDL-C levels, respectively, and all these differences were statistically significant (*p* < 0.01). Further, HDL-C levels increased by 33.87, 51.48, and 56.11%, respectively, and these increases were significant and dose dependent (*p* < 0.01). Compared with the blank group, the effect values of TC, TG, LDL-C, and HDL-C in the model group were 2.073, 3.943, 1.498, and 3.469, respectively. Compared with the model group, the effect values of TC in the low-, medium-, and high-dose groups were 0.034, 0.611, and 1.961, respectively. Compared with the model group, the effect values of TG in the low-, medium-, and high-dose groups were 0.642, 1.941, and 2.274, respectively. Compared with the model group, the effect values of LDL-C in the low-, medium-, and high-dose groups were 0.147, 0.341, and 0.675, respectively. Compared with the model group, the effect values of HDL-C in the low-, medium-, and high-dose groups were 1.503, 2.005, and 2.835, respectively. The results of this study indicated that high-dose naringin can effectively inhibit increases and decreases in LDL-C and HDL-C levels, respectively, in a high-fat HepG2 cell model, thus promoting cholesterol metabolism. The in vitro lipid-lowering test method is simple, has a short cycle, and has relatively few influencing factors. One of the important methods for in vitro lipid-lowering activity is to determine the binding ability of the sample to bile salts. Bile acids are divided into free and bound bile acids, which are synthesized by the liver and flow into the intestine with bile secretions in the bile ducts. Free bile acids undergo passive reabsorption combined with active reabsorption to form a complete hepatic intestinal circulation system and regulate the bile acid content in the body to maintain a balanced and stable level. Studies have shown that flavonoids can bind with bile salts, reduce the reabsorption of bile acids, promote the degradation of cholesterol accumulated in tissues such as the liver, synthesize more bile salts, and lower the cholesterol content in the body, achieving a lipid-lowering effect. Research has shown that the adsorption and binding mechanisms of flavonoids include hydrophobic interactions, electrostatic interactions, and fluid dynamics, which are closely related to their total sugar content and monosaccharide composition. Flavonoids and bile acid binding sites are related to rhamnose. The structure of naringin dihydrochalcone is a complex of naringin, rhamnose, and glucose which can increase the binding rate with bile salts. However, there have been few studies on the in vitro lipid-lowering effect of naringin dihydrochalcone. This study focuses on pancreatic lipase, the most important enzyme for hydrolyzing dietary fat; bile salts, which are mainly concentrated in human and animal bile and play an important role in the metabolism and absorption of lipids; cholesterol; fat-soluble vitamins; and other substances. HepG2, a cell with important lipid metabolism physiological functions, was evaluated for in vitro lipid-lowering effects based on simulating the gastrointestinal environment and constructing a high-cholesterol cell model. The rate of inhibition of pancreatic lipase by naringin dihydrochalcone, the binding ability of two typical bile salts, and the lipid-lowering activity of HepG2 in a high-cholesterol cell model were tested to provide a theoretical basis and data support for the development of naringin functional products.

## 3. Materials and Methods

### 3.1. Materials and Reagents

This test uses pomelo peel (maturity 50%) as the raw material, and it came from Shatian Village, Songshan Town, Rongxian County, Guangxi Province.

Naringin (purity > 98%) was obtained from Hefei Bomei Biotechnology Co., Ltd. (Hefei, China).

Sodium glycolic acid (purity > 98%) was obtained from Hefei Qiansheng Biotechnology Co., Ltd. (Hefei, China).

D101 macroporous resin, sodium taurocholate, trypsin, and pepsin were acquired from Solebo Biotechnology Co., Ltd. (Beijing, China).

Chromatography-grade methanol was purchased from Tianjin Guangfu Reagent Co., Ltd. (Tianjin, China).

Naringin dihydrochalcone standard (purity > 98%) was purchased from Xi’an Huilin Biological Products Co., Ltd. (Xi’an, China).

Palladium carbon was purchased from Tianjin Baima Technology Co., Ltd. (Tianjin, China).

Spectral-grade deuteroacetone was purchased from Shanghai Yien Chemical Technology Co., Ltd. (Shanghai, China).

Spectral-grade potassium bromide was purchased from Tianjin Hengchuang Lida Technology Development Co., Ltd. (Tianjin, China).

Spectral-grade tetramethylsilane was purchased from Hangzhou Sloan Material Technology Co., Ltd. (Hangzhou, China).

HepG2 cells were purchased from the Shanghai Institute of Biology, Chinese Academy of Sciences. (Shanghai, China).

Cholesterol (purity > 99%) was purchased from Sigma Chemical Co., Ltd. (St. Louis, MO, USA).

Total cholesterol (Cat. No. A111-2-1), triglyceride (Cat. No. A110-1-1), low-density lipoprotein cholesterol (Cat. No. A113-1-1), and high-density lipoprotein cholesterol (Cat. No. A112-1-1) were obtained from the Nanjing Jiancheng Bioengineering Institute (Nanjing, China).

### 3.2. Optimization of the Naringin Extraction Process

#### 3.2.1. Pomelo Peel Pretreatment

At a room temperature of 25 °C, the yellow outer skin of the pomelo peel was removed to obtain a white, sponge-shaped pomelo sac. The pomelo sac was dried at constant temperature and humidity in an oven at 50 °C and was crushed through an 80 mesh sieve. The pomelo peel was sealed in a packaging bag and placed in a dryer maintained at 4 °C until further use [33].

#### 3.2.2. Establishment of a Naringin Standard Curve

At a room temperature of 25 °C, an 80 mg sample of naringin standard was accurately weighed and diluted with absolute ethanol in a 500 mL volumetric flask to obtain a 0.16 mg/mL naringin standard solution. Different volumes of the solution were then distributed into 10 mL colorimetric tubes (0, 0.3, 0.6, 0.9, 1.2, 1.5, and 1.8 mL). Next, 5 mL of absolute ethanol and 0.1 mL of 4 M NaOH solution were successively added to each tube [34,35]. Thereafter, distilled water was added to scale, and the samples were shaken thoroughly and placed in a water bath maintained at 40 °C for 10 min. After rapid cooling, absorbance measurements were performed at 420 nm (UV Visible Spectrophotometer, UV-6300, Meipeda Instrument Co., Ltd., Shanghai, China). The average values of three replicates for each concentration were obtained. These data were then used to construct a naringin standard curve [36,37].

#### 3.2.3. Calculation of Extraction Yield of Naringin

Pomelo peel powder (5 g) was accurately weighed (W) and was extracted with absolute ethanol. The filtrate thus obtained was placed in a 500 mL volumetric flask [38]. We measured the absorbance value according to the method in Section 3.2.2, corresponding to the standard curve of naringin, calculated the concentration of the diluent, calculated the extraction yield according to the following Formula (3), set three equilibria, and took the average value [39].

Naringinextraction yield (mg/g) = CV/W (3)

where C—concentration of naringin in diluent (mg/mL);

V—volume of extract (mL);

W—mass of pomelo peel powder (g).

#### 3.2.4. Single-Factor Experimental Design

At a room temperature of 25 °C, naringin powder (0.5 g) was accurately weighed, and the effects of different solid–liquid ratios, extraction times, and extraction temperatures on its extraction yield were investigated to determine the behaviour of its surface under the influence of each factor. An ethanol volume fraction of 75%, feed-to-solvent ratio of 45:1 mL/g, extraction time of 2.0 h, and extraction temperature of 60 °C were used as the basic conditions for the singl1-factor screening test. When studying a given factor, all the other conditions were kept constant. Flavonoids are a class of natural products with multiple hydroxyl groups and good solubility. Ethanol is a commonly used polar solvent with good solubility for many natural products. During the extraction process, ethanol can effectively dissolve flavonoids and separate them from the raw materials [40].

#### 3.2.5. Response Surface Experiment Design

On the basis of single-factor experiment, according to the principle of Box–Behnken center combination design, the optimal extraction range was selected, with naringin concentration as the response value; the extraction temperature (X_1_), solid–liquid ratio (X_2_), and extraction time (X_3_) as independent variables; and the extraction yield of naringin as the response value [41]. Data analysis was carried out using the Design-Expert 8.0.6 software, and an optimization model of three factors and three levels was obtained, totaling 17 groups of experiments [42].

### 3.3. Purification of Naringin

At a room temperature of 25 °C, we purified the crude naringin product prepared in the above experiment using DM101 macroporous resin, and the purification conditions were determined to be a loading concentration of 0.075 mg/mL, a sample solution pH of 3.5, and a loading flow rate was 1.5 mL/min [43].

### 3.4. Structural Identification of Naringin

At a room temperature of 25 °C, the Nicolet iS5 Fourier transform infrared spectrometer was used for sample infrared spectroscopy (FT-IR) detection. The potassium bromide tablet method was used for sample preparation. The sample was mixed evenly with potassium bromide and ground and pressed in an agate mortar [44,45,46]. The scanning range was from 500 cm^−1^ to 4000 cm^−1^. An AVANCE III nuclear magnetic resonance spectrometer was used to detect the nuclear magnetic resonance hydrogen spectrum (^1^H NMR) and carbon spectrum (^13^C NMR) of the sample. Solvent: deuterated acetone; internal standard: tetramethylsilane (TMS) [47,48,49].

### 3.5. Preparation of Naringin Dihydrochalcone

At a room temperature of 25 °C, purified naringin (5.0 g) was placed in a stainless steel high-pressure reactor. Thereafter, NaOH solution was slowly added to obtain a reaction solution with pH 14. Next, excess 10% palladium carbon catalyst was also added, and the pressure and temperature of the reactor were maintained at 1.6 MPa and 50 °C, respectively. The reactor was then placed on a magnetic stirrer, and hydrogen gas was introduced for 3 h before the reaction commenced. After 1 h, the hydrogen valve was closed to maintain the reaction rate. After 12 h, when the reaction reached completion, the product was filtered to recover the catalyst. Then, the filtrate was removed from the NaOH solution using 732 cation exchange resin, and the hydrogenation product was obtained after standing, filtering, and drying [50]. Under these conditions, the obtained yield of naringin dihydrochalcone was 92.35%.

### 3.6. Structural Identification of Naringin Dihydrochalcone

At a room temperature of 25 °C, the Nicolet iS5 Fourier transform infrared spectrometer was used for sample infrared spectroscopy (FT-IR) detection. The potassium bromide tablet method was used for sample preparation. The sample is mixed evenly with potassium bromide and ground and pressed in an agate mortar. The scanning range was from 500 cm^−1^ to 4000 cm^−1^. An AVANCE III nuclear magnetic resonance spectrometer was used to detect the nuclear magnetic resonance hydrogen spectrum (^1^H NMR) and carbon spectrum (^13^C NMR) of the sample. Solvent: dimethyl sulfoxide (DMSO); internal standard: tetramethylsilane (TMS).

### 3.7. In Vitro Study of the Effects of Naringin Dihydrochalcone on Lowering Blood Lipid Contents

#### 3.7.1. Drawing the Standard Curve of Cholic Acid Salt

At a room temperature of 25 °C, standard solutions of sodium glycocholic acid and sodium taurocholate at concentrations of 0.05, 0.10, 0.15, 0.20, and 0 were prepared. Standard solutions (2 mL) of different concentrations were placed in different test tubes [51]. Next, 8 mL of a 60% H_2_SO_4_ solution was added, and the mixture was heated in a water bath at 80 °C for 10 min. Thereafter, the mixture was placed in an ice bath for 5 min. This was followed by absorbance measurements at 387 nm [52].

#### 3.7.2. Naringin Dihydrochalcone Binding Cholate Experiment

Naringin dihydrochalcone solutions were prepared with concentrations of 100, 200, 300, 400, and 500 mg/L at a room temperature of 25 °C. Next, 2 mL of each concentration solution were taken, and 3 mL of 10 mg/mL pepsin solution was added followed by 1 mL of 0.01 mol/L HCl solution. This was then digested in a 37 °C incubator for 1 h to simulate the gastric environment. Next, 6 mL of 10 mg/mL trypsin solution were added separately, the pH was adjusted to 6.3 with 0.1 mol/L NaOH solution, and the result was digested in a constant-temperature shaking incubator at 37 °C for 1 h to simulate the intestinal environment. Afterward, 6 mL of sodium taurocholate solution and sodium glycocholate solution with a concentration of 0.4 mmol/L, respectively, was adder, and the mixture was shaken at constant temperature (37 °C,1 h), centrifuged (4500 r/min, 15 min), and the supernatant was taken. Finally, the absorbance was measured at a wavelength of 387 nm; The process was repeated three times.

Each sample was measured three times in parallel, and the remaining content of glycylcholic acid and taurocholate was calculated according to the standard curve. The ratio of the difference between the total amount of glycocholate or taurocholate added and the remaining amount to the total amount is the binding rate, expressed as a percentage [53].

#### 3.7.3. Determination of Conjugation Rate of Cholic Acid Salts

The ability of naringin dihydrochalcone to lower blood lipid levels was determined based on the binding rate of the two sodium cholates with naringin dihydrochalcone [54].

#### 3.7.4. MTT Cell Proliferation and Toxicity Experiments

At a room temperature of 25 °C, we used the MTT colorimetric method to detect the effects of different concentrations of naringin dihydrochalcone on the proliferation of HepG2 cells. For cell digestion (1–2 min), 0.25% trypsin was added to HepG2 cells at the logarithmic growth stage cultured in MEM medium containing 1% dual antibody and 10% foetal bovine serum at 37 °C in a 5% CO_2_ incubator. Thereafter, the cells were counted, inoculated into a 96-well plate (100 cells per well), and incubated at 37 °C in a 5% CO_2_ atmosphere for 24 h until the cell count reached 5000 cells per well. New culture media with the naringin dihydrochalcone at different concentrations (6.25, 12.50, 25.00, 50.00, 100.00, 200.00, 400.00, 800.00, and 1600.00 μg/mL) were added to the cell culture followed by further incubation for 24 h. Thereafter, the supernatant was discarded, and the residue was incubated with 5 mg/mL MTT solution for 4 h, after which 100 μL of dimethyl sulfoxide (DMSO) solution was added. Further, after the crystal violet staining at the bottom of the incubator disappeared completely, the cells were inoculated into a 96-well plate and the optical density was measured at wavelength of 490 nm using a microplate reader (BioTek, SYNERGYH1, Agilent Co., Ltd., Santa Clara, CA, USA) [55]. Formula (4) for calculating cell survival rate was as follows:

(4) $$\mathrm{Cell}\;\mathrm{survival}\;\mathrm{rate}\;=\frac{Dosing\;group\;absorbance\;value}{Control\;group\;absorbance\;value}\times 100\%$$

#### 3.7.5. High-Cholesterol Cell Model

At a room temperature of 25 °C, when HepG2 liver cancer cells covered 80–90% of the bottom of the cell incubation bottle, the cells were digested with tripsin, suspended, and counted. Thereafter, the cells at a concentration of 2 × 10^5^ cells/mL were inoculated into a 96-well plate and incubated in a 5% CO_2_ incubator for 24–48 h. When the cell monolayer reached the bottom of the well plate, the culture medium was replaced with serum-free medium, and cholesterol solutions of different mass concentrations (10, 15, 20, 25, and 30 μg/mL) were added. After incubation for 24 h, the culture medium was discarded, and the cells were washed three times with phosphate-buffered saline (PBS). The HepG2 cells were further digested using trypsin, and the cell suspension obtained was transferred into a centrifuge tube. Centrifugation was performed at 1500 r/min for 4 min. After this step, the supernatant was discarded and the residue was washed with PBS. Centrifugation was further performed, and the resulting supernatant was discarded. The cell residue was then resuspend in PBS and crushed using a cell crusher. The concentration of cholesterol in each cell group was then determined in accordance with the instructions provided with the reagent kit [56].

#### 3.7.6. Effects of Naringin Dihydrochalcone on TG, TC, LDL-C, and HDL-C Levels in HepG2 Cells

At a room temperature of 25 °C, when HepG2 cells in the cell culture bottle covered 80–90% of the bottom of the bottle, the cells were digested with trypsin, centrifuged, and diluted to a given concentration. This was followed by inoculation into a 96-well plate; the addition of 2.5 mL cholesterol at 10, 20, and 40 mg/kg to the complete culture medium; and incubation for 24 h. The supernatant was aspirated, and the residue was digested using trypsin, was centrifuged, and was washed with PBS three times. Finally, 250 μL of 1% TritonX-100 was added followed by cracking in an ice bath at 4 °C for 30–40 min and the detection of TG, TC, LDL-C, and HDL-C levels according to instructions stated in the reagent kits.

### 3.8. Data Processing

The test data were analysed using Design-Expert 8.0.6 and Origin 2021 software.

## 4. Conclusions

In this study, optimal conditions naringin extraction from pomelo wastes were determined (67 °C, 2.8 h, and 54:1 mL/g). Additionally, the naringin dihydrochalcone obtention was realized and characterized by spectroscopic methods. Further, the extraction efficiency of naringin was 3.248%, and via IR and NMR, its structure as well as that of naringin dihydrochalcone were confirmed. The determination of the lipid-lowering activity of naringin dihydrochalcone in vitro was also investigated. Specifically, we used cholesterol induction to establish a high-fat HepG2 cell model, and compared with the model group, naringin dihydrochalcone significantly reduced intracellular TC and TG levels, indicating that it exerts a cholesterol metabolism-promoting effect and can effectively prevent lipid deposition in blood vessels and the liver, while also showing a lipid-lowering effect. Hyperlipidaemia, as a disorder of lipid metabolism, poses a serious threat to human health. The increase of TG level is the key factor to induce cardiovascular diseases such as atherosclerosis, coronary heart disease, and stroke. Based on this, we established a high-cholesterol model of HepG2 cells and conducted toxicity experiments on HepG2 cells, proving that naringin has good compatibility with HepG2 cells. Cholesterol is a lipophilic substance that can bind with proteins to form lipoproteins and dissolve in the bloodstream. Specifically, low-density lipoprotein cholesterol easily adheres to the blood vessel wall, leading to an increase in blood lipid levels. Naringin can effectively reduce low-density lipoprotein levels. These findings indicate that naringin dihydrochalcone has the potential for application as a functional food resource for reducing blood lipid levels. In terms of future prospects, we hope to further explore the intervention effect of naringin dihydrochalcone on the intestinal microenvironment based on the preliminary research on the lipid-lowering effect of naringin dihydrochalcone in this article, especially revealing the mechanism of naringin dihydrochalcone in regulating intestinal microbiota and reducing cholesterol.

## Figures and Tables

**Table 1:** Response surface optimization test results.

Test Number	X_1_ (Extraction Temperature)	X_2_ (Feed to Solvent Ratio)	X_3_ (Extraction Time)	Extraction Efficiency %
1	0	0	0	3.171
2	0	−1	1	3.015
3	−1	−1	0	2.945
4	0	0	0	3.174
5	−1	0	1	3.137
6	0	0	0	3.191
7	−1	1	0	3.130
8	0	−1	−1	2.870
9	1	0	1	3.186
10	−1	0	−1	2.888
11	1	1	0	3.202
12	1	−1	0	3.090
13	0	0	0	3.188
14	0	0	0	3.177
15	1	0	−1	2.972
16	0	1	−1	2.945
17	0	1	1	3.241

**Table 2:** Response surface optimisation test results.

Source	Sum of Squares	Degrees of Freedom	Mean Square	*f* Value	*p* Value	Significance
Model	23.89	9	2.65	148.02	<0.0001	**
X_1_	1.53	1	1.53	85.39	<0.0001	**
X_2_	4.47	1	4.47	249.27	<0.0001	**
X_3_	10.22	1	10.22	569.64	<0.0001	**
X_1_ X_2_	0.13	1	0.13	7.43	0.0295	*
X_1_ X_3_	0.031	1	0.031	1.71	0.2326	
X_2_ X_3_	0.57	1	0.57	31.79	0.0008	**
X_1_^2^	0.38	1	0.38	21.45	0.0024	**
X_2_^2^	1.43	1	1.43	79.60	<0.0001	**
X_3_^2^	4.57	1	4.57	255.05	<0.0001	**
residual	0.13	7	0.018			
Spurious term	0.094	3	0.031	4.05	0.1050	
Error term	0.031	4	0.00777			
the sum	24.02	16				

Note: * indicates significant difference, *p* < 0.05; ** Indicates that the difference is extremely significant, *p* < 0.01.

## Data Availability

Data are contained within the article and Supplementary Materials.

## Supplementary Materials

The following supporting information can be downloaded at: https://www.mdpi.com/article/10.3390/molecules29235778/s1, Figure S1: The standard curve of naringin; Figure S2: Effects of different extraction parameters on the naringin extraction rate; Figure S3: Fourier transform infrared spectrum of naringin; Figure S4: 1HNMR spectrum of naringin products; Figure S5: 13C NMR spectrum of naringin products; Figure S6: 1HNMR spectrum of naringin standard; Figure S7: 13C NMR spectrum of naringin standard; Figure S8: Fourier transform infrared spectrum of naringin dihydrochalcone; Figure S9: 1HNMR spectrum of refined naringin dihydrochalcone products; Figure S10: 13C NMR spectrum of refined naringin dihydrochalcone products; Figure S11: 1H NMR spectrum of naringin dihydrochalcone standard; Figure S12: 13C NMR spectrum of naringin dihydrochalcone standard; Figure S13: Standard curve of sodium glycocholate; Figure S14: Standard curve of sodium taurocholate.
