# Supplementary material for: Optimization of Naringin Extraction, Synthesis of Dihydrochalcone and Its Effects on Reducing Blood Lipid Levels In Vitro
Source: Molecules. 2024 Dec 6;29(23):5778. doi: 10.3390/molecules29235778 (PMC11643946; doi:10.3390/molecules29235778)
Supplement: Supplementary file 1 [file molecules-29-05778-s001.zip › molecules-3274760-supplementary.pdf]

Supplementary Material

Extraction of naringin dihydrochalcone and its effect on reducing blood lipid levels in vitro

Xiao-lei Yu[1, 2,*],Hao-wei Wu[1] , Lei Zhang[2,*] and Dong-liang Fei[1,*]

[1]Jinzhou Medical University, Jinzhou 121000, China

[2]MOE Key Laboratory for Nonequilibrium Synthesis and Modulation of Condensed Matter, School of Physics, Xi'an Jiaotong University, Xi'an 710049, China

*** Correspondence: Corresponding Author:** zhangleio@mail.xjtu.edu.cn; Tel.: +86-029-8266-8634; feidongliang@jzmu.edu.cn; Tel.: +86-0416-3675-165

Figures captions

Fig. S1. The standard curve of naringin.

Fig. S2. Effects of different extraction parameters on the naringin extraction rate.

Fig. S3. Fourier transform infrared spectrum of naringin.

Fig. S4. ^{1}HNMR spectrum of naringin products.

Fig. S5. ^{13}C NMR spectrum of naringin products.

Fig. S6. ^{1}HNMR spectrum of naringin standard.

Fig. S7. ^{13}C NMR spectrum of naringin standard.

Fig. S8. Fourier transform infrared spectrum of naringin dihydrochalcone.

Fig. S9. ^{1}HNMR spectrum of refined naringin dihydrochalcone products.

Fig. S10. ^{13}C NMR spectrum of refined naringin dihydrochalcone products.

Fig. S11. ^{1}H NMR spectrum of naringin dihydrochalcone standard.

Fig. S12. ^{13}C NMR spectrum of naringin dihydrochalcone standard.

Fig. S13. Standard curve of sodium glycocholate.

Fig. S14. Standard curve of sodium taurocholate

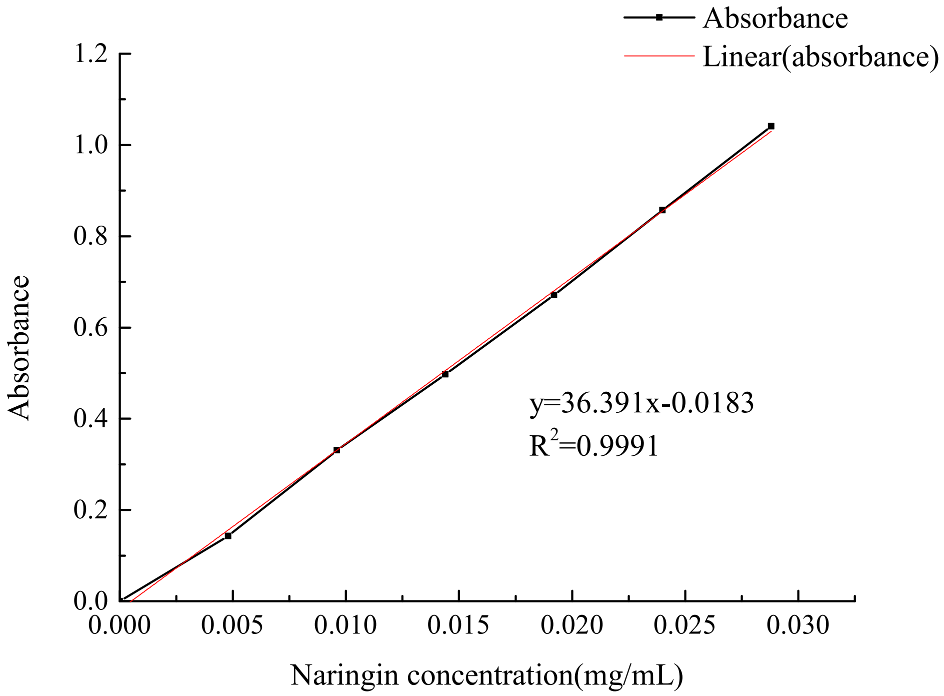

Fig. S1. The standard curve of naringin.

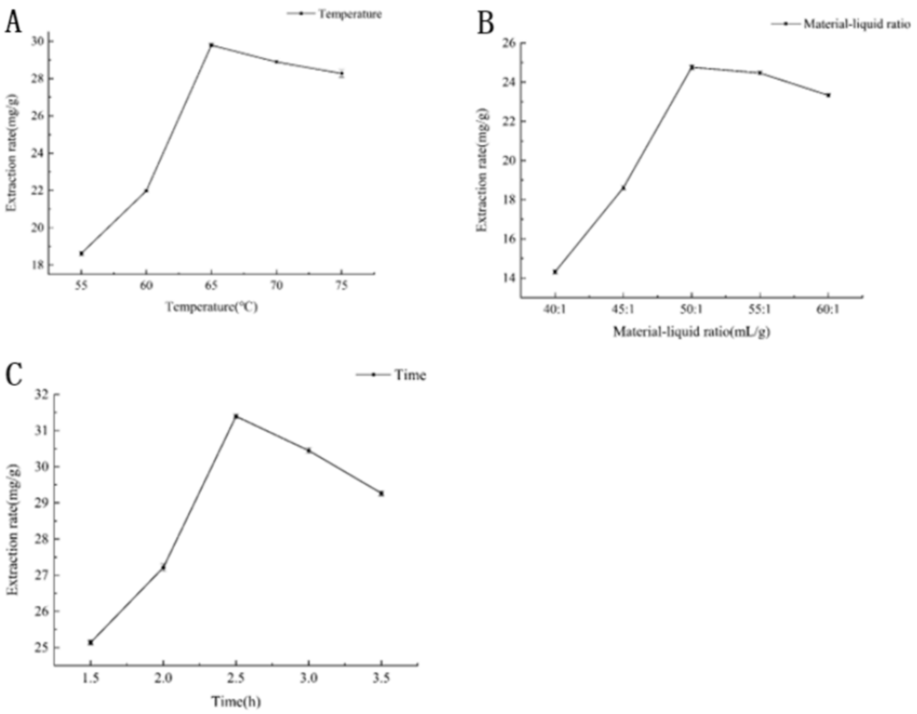

Fig.S2. Effects of different extraction parameters on the naringin extraction rate. （A）The effect of extraction temperature on the extraction rate of naringin;（B）The effect of material-liquid ratio on the extraction rate of naringin;（C）The effect of extraction time on the extraction rate of naringin.

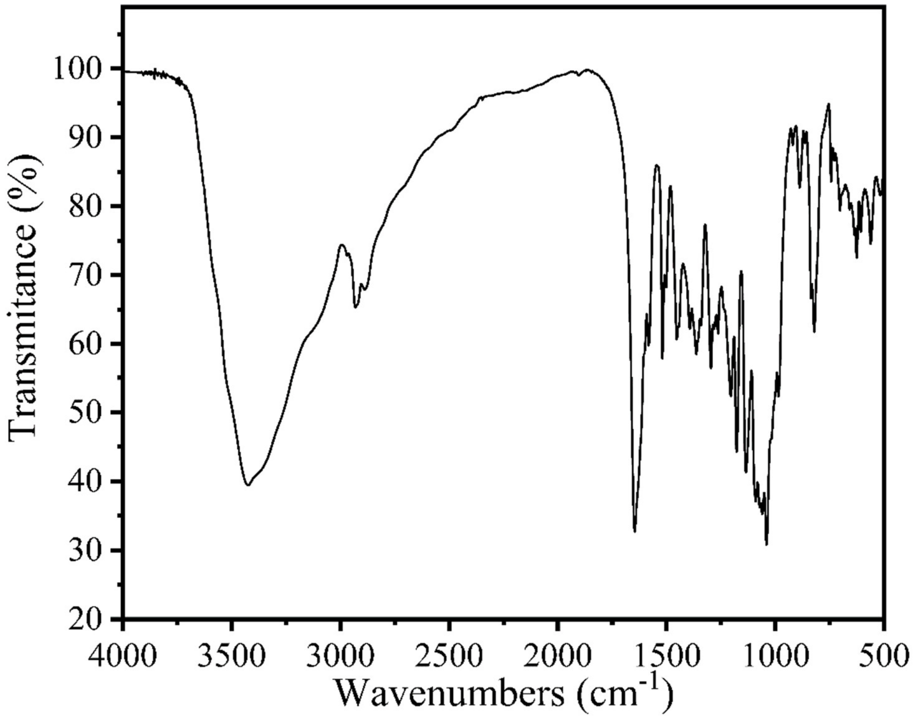

Fig. S3. Fourier transform infrared spectrum of naringin

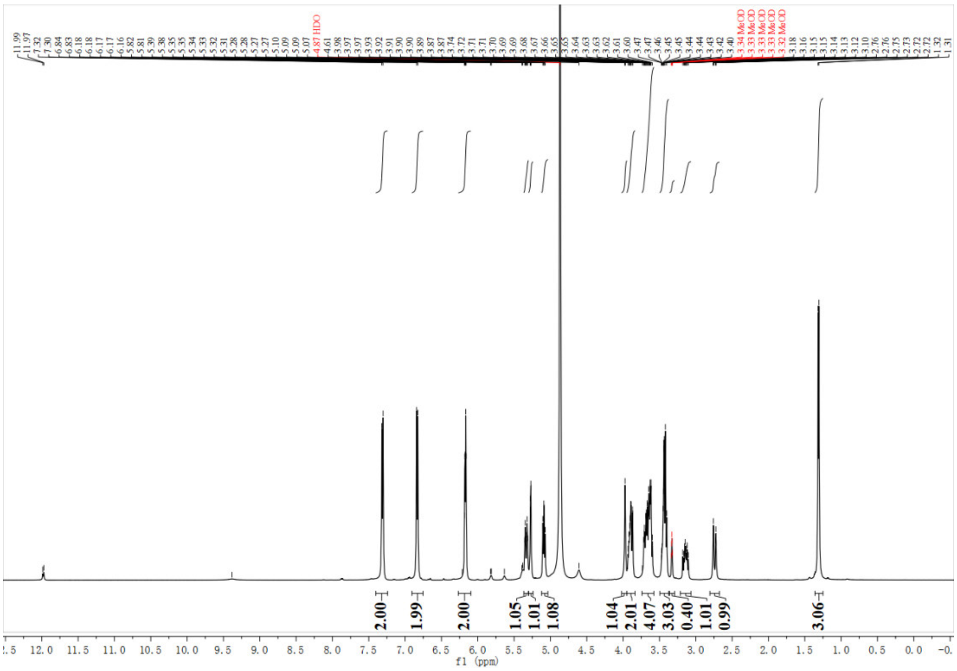

Fig. S4. ^{1}HNMR spectrum of naringin products.

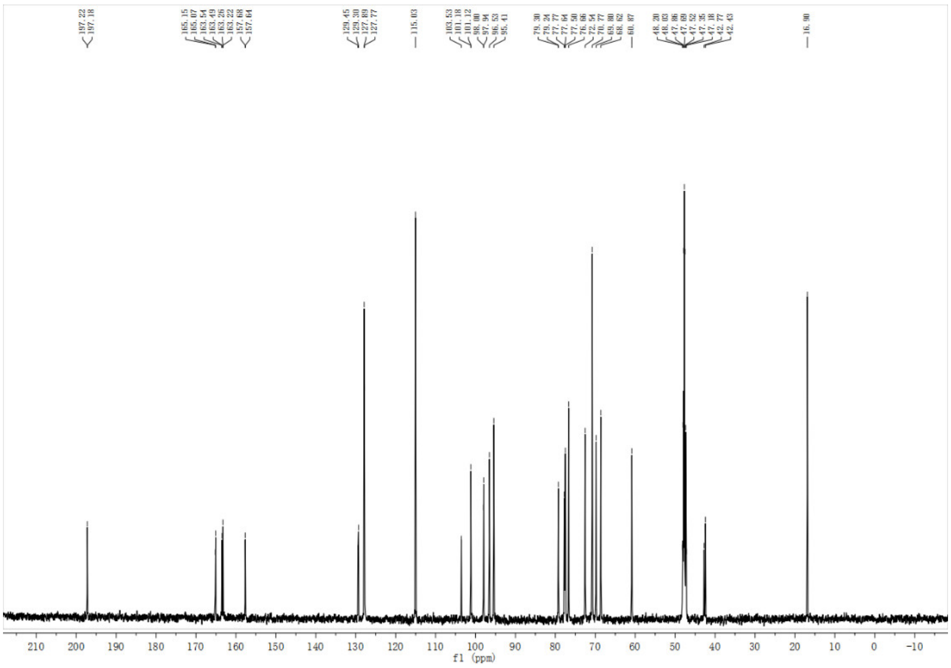

Fig. S5. ^{13}C NMR spectrum of naringin products.

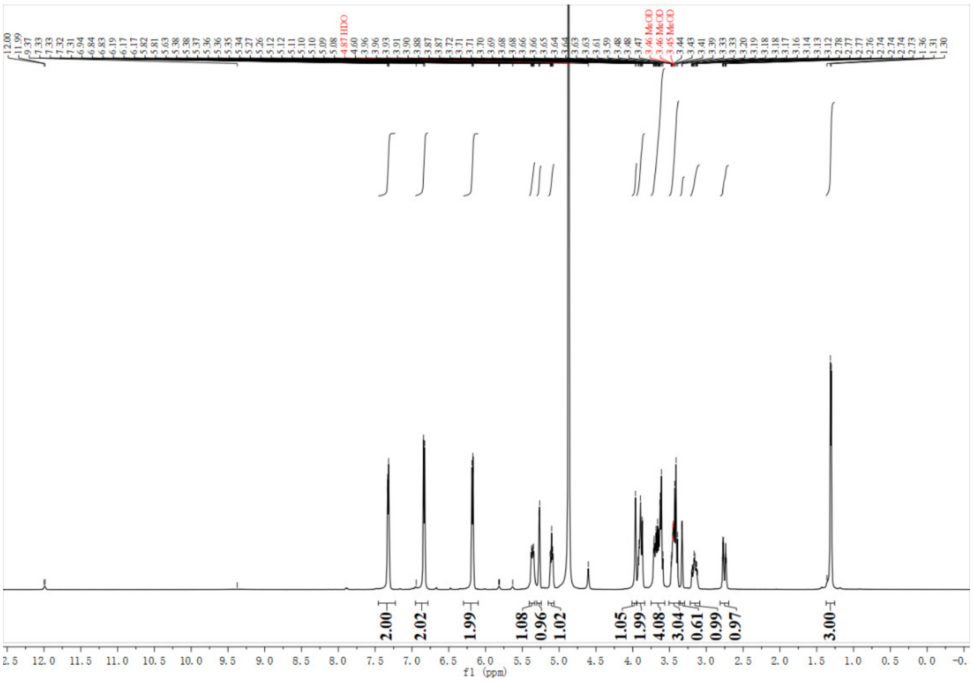

Fig. S6. ^{1}HNMR spectrum of naringin standard.

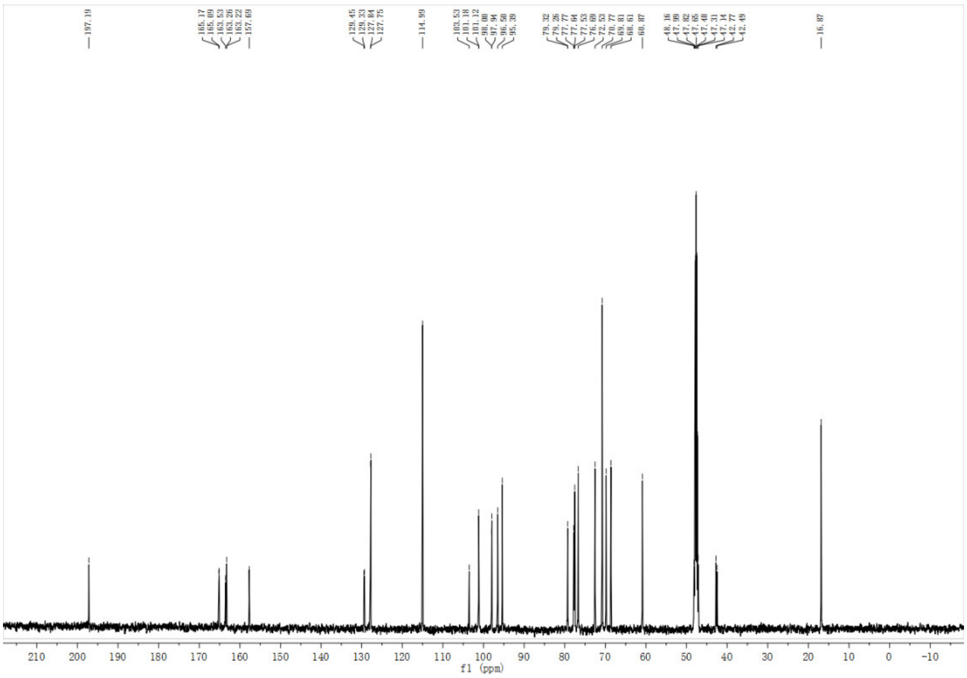

Fig. S7. ^{13}C NMR spectrum of naringin standard.

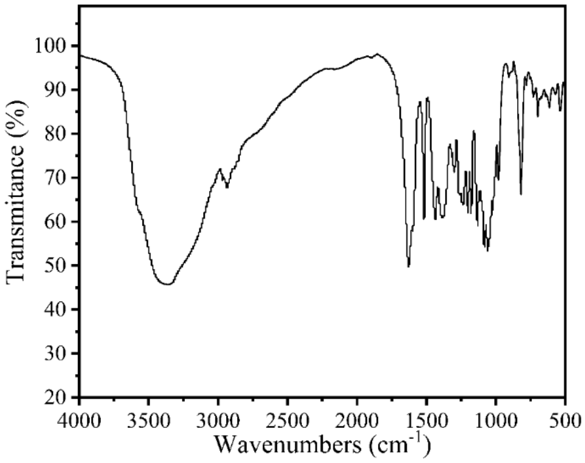

Fig. S8. Fourier transform infrared spectrum of naringin dihydrochalcone.

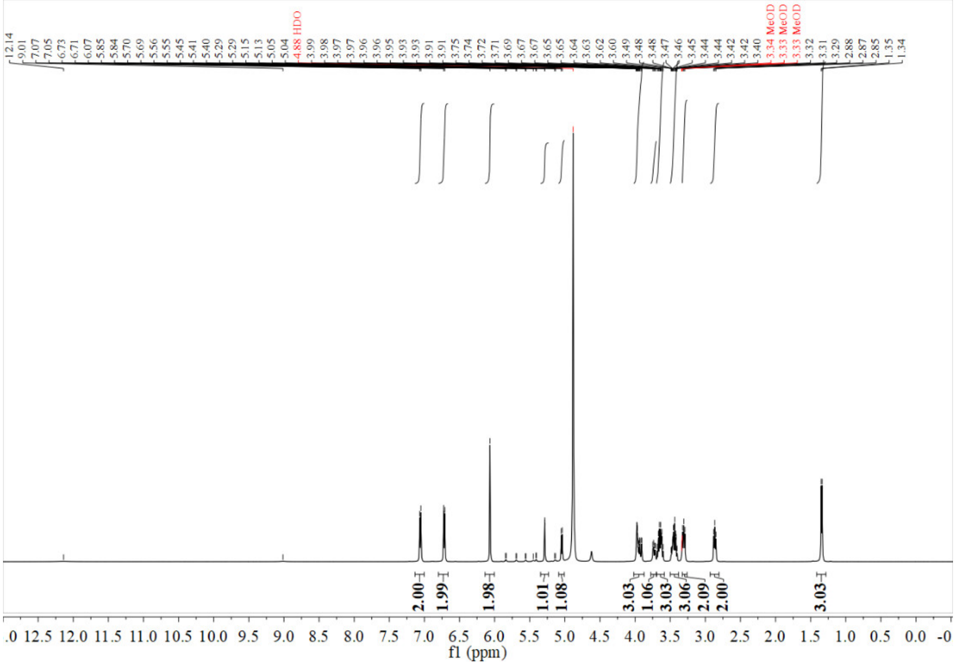

Fig. S9. ^{1}HNMR spectrum of refined naringin dihydrochalcone products.

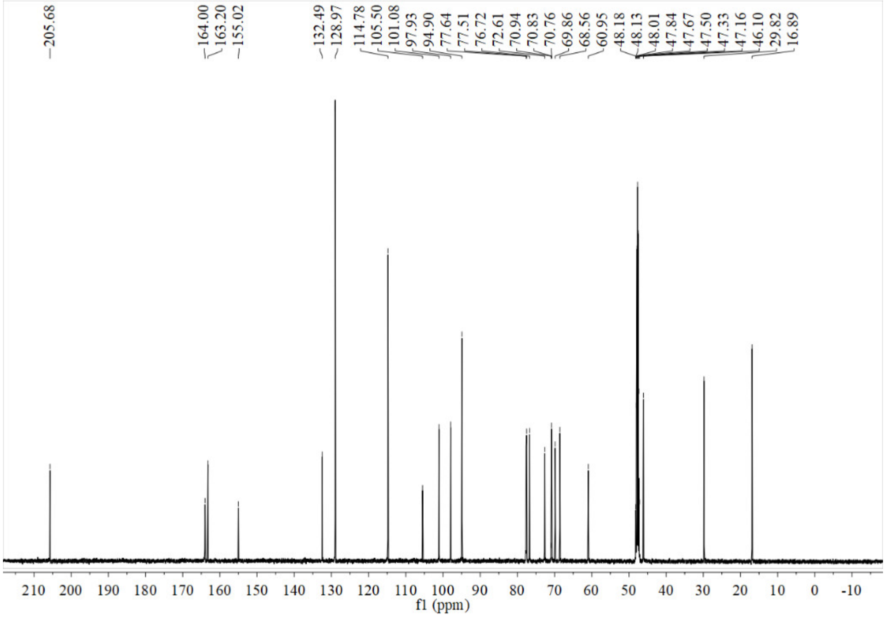

Fig. S10. ^{13}C NMR spectrum of refined naringin dihydrochalcone products.

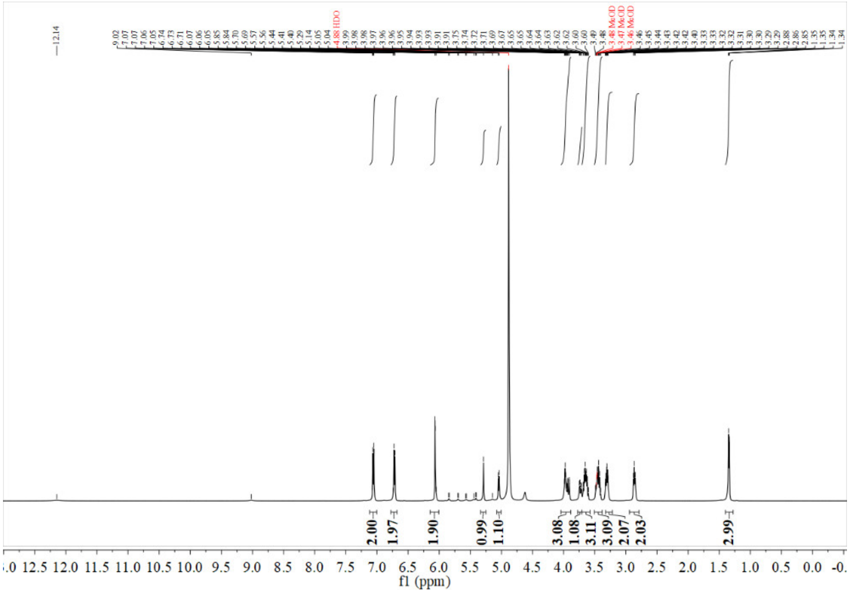

Fig. S11. ^{1}H NMR spectrum of naringin dihydrochalcone standard.

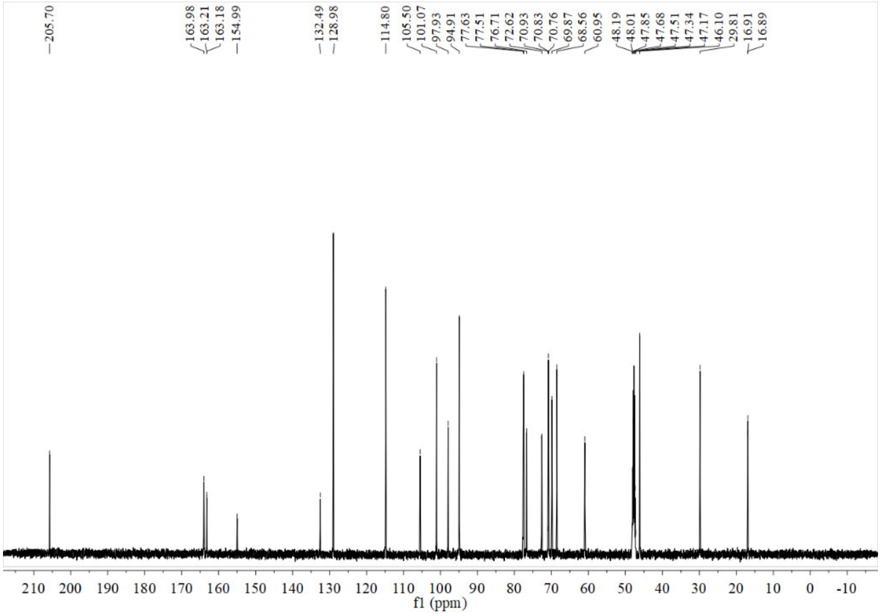

Fig. S12. ^{13}C NMR spectrum of naringin dihydrochalcone standard.

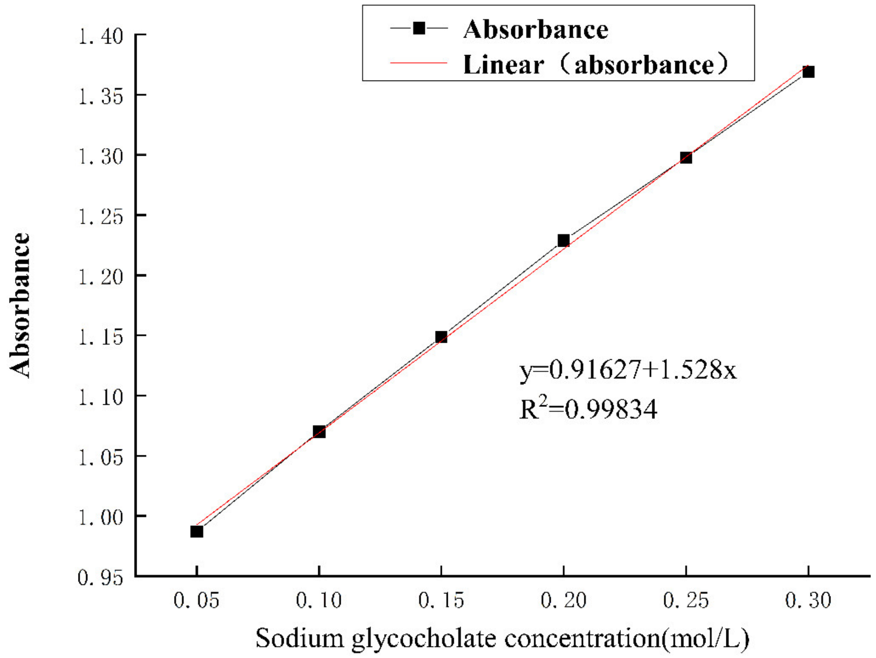

Fig. S13. Standard curve of sodium glycocholate.

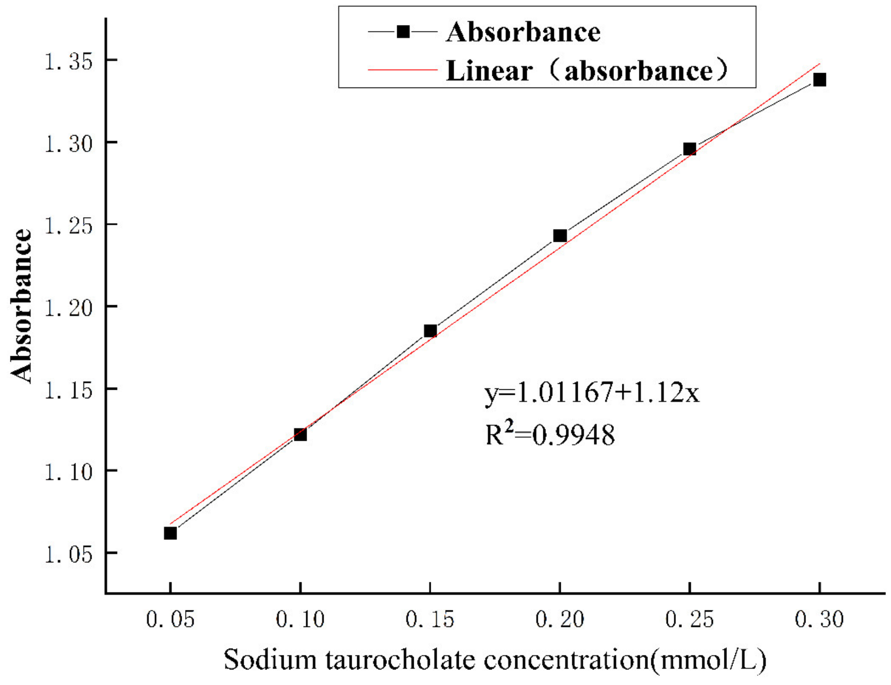

Fig. S14. Standard curve of sodium taurocholate
